# Supplementary material for: HIVprotI: an integrated web based platform for prediction and design of HIV proteins inhibitors
Source: J Cheminform. 2018 Mar 9;10:12. doi: 10.1186/s13321-018-0266-y (PMC5845081; doi:10.1186/s13321-018-0266-y)
Supplement: Supplementary file 2 — Additional file 2. Source code of HIVProtI web server. [file 13321_2018_266_MOESM2_ESM.zip › HIVprotI_Source-code/hivprotI/weka-3-6-6/doc/weka/core/xml/XMLDocument.html]

XMLDocument


---


|  |  |  |
| --- | --- | --- |
| **Overview** \| **Package** \| **Class** \| **Tree** \| **Deprecated** \| **Index** \| **Help** \| **Weka's home** | |  |
| **PREV CLASS**   **NEXT CLASS** | **FRAMES**    **NO FRAMES**     **All Classes** |  |
| SUMMARY: NESTED \| FIELD \| CONSTR \| METHOD | DETAIL: FIELD \| CONSTR \| METHOD |  |


---


## weka.core.xml Class XMLDocument

```
java.lang.Object
  weka.core.xml.XMLDocument
```

**All Implemented Interfaces:**: RevisionHandler

**Direct Known Subclasses:**: XMLInstances

---

``` public class XMLDocument extends java.lang.Object implements RevisionHandler ```

This class offers some methods for generating, reading and writing
XML documents.  
It can only handle UTF-8.

**Version:**
:   $Revision: 1.9 $

**Author:**
:   FracPete (fracpete at waikato dot ac dot nz)

**See Also:**: `PI`

---

| **Field Summary** | |
| --- | --- |
| `static java.lang.String` | `ATT_NAME`             the "name" attribute. |
| `static java.lang.String` | `ATT_VERSION`             the "version" attribute. |
| `static java.lang.String` | `DTD_ANY`             the ANY placeholder. |
| `static java.lang.String` | `DTD_AT_LEAST_ONE`             the at least one marker. |
| `static java.lang.String` | `DTD_ATTLIST`             the AttList definition. |
| `static java.lang.String` | `DTD_CDATA`             the CDATA placeholder. |
| `static java.lang.String` | `DTD_DOCTYPE`             the DocType definition. |
| `static java.lang.String` | `DTD_ELEMENT`             the Element definition. |
| `static java.lang.String` | `DTD_IMPLIED`             the #IMPLIED placeholder. |
| `static java.lang.String` | `DTD_OPTIONAL`             the optional marker. |
| `static java.lang.String` | `DTD_PCDATA`             the #PCDATA placeholder. |
| `static java.lang.String` | `DTD_REQUIRED`             the #REQUIRED placeholder. |
| `static java.lang.String` | `DTD_SEPARATOR`             the option separator. |
| `static java.lang.String` | `DTD_ZERO_OR_MORE`             the zero or more marker. |
| `static java.lang.String` | `PI`             the parsing instructions "<?xml version=\"1.0\" encoding=\"utf-8\"?>" (may not show up in Javadoc due to tags!). |
| `static java.lang.String` | `VAL_NO`             the value "no". |
| `static java.lang.String` | `VAL_YES`             the value "yes". |


**Constructor Summary**

`XMLDocument()`
  
          initializes the factory with non-validating parser.

`XMLDocument(java.io.File file)`
  
          Creates a new instance of XMLDocument.

`XMLDocument(java.io.InputStream stream)`
  
          Creates a new instance of XMLDocument.

`XMLDocument(java.io.Reader reader)`
  
          Creates a new instance of XMLDocument.

`XMLDocument(java.lang.String xml)`
  
          Creates a new instance of XMLDocument.


| **Method Summary** | |
| --- | --- |
| `void` | `clear()`             sets up an empty DOM document, with the current DOCTYPE and root node. |
| `java.lang.Boolean` | `evalBoolean(java.lang.String xpath)`             Evaluates and returns the boolean result of the XPath expression. |
| `java.lang.Double` | `evalDouble(java.lang.String xpath)`             Evaluates and returns the double result of the XPath expression. |
| `java.lang.String` | `evalString(java.lang.String xpath)`             Evaluates and returns the boolean result of the XPath expression. |
| `org.w3c.dom.NodeList` | `findNodes(java.lang.String xpath)`             Returns the nodes that the given xpath expression will find in the document. |
| `javax.xml.parsers.DocumentBuilder` | `getBuilder()`             returns the DocumentBuilder. |
| `static java.util.Vector` | `getChildTags(org.w3c.dom.Node parent)`             returns all non tag-children from the given node. |
| `static java.util.Vector` | `getChildTags(org.w3c.dom.Node parent, java.lang.String name)`             returns all non tag-children from the given node. |
| `static java.lang.String` | `getContent(org.w3c.dom.Element node)`             returns the text between the opening and closing tag of a node (performs a `trim()` on the result). |
| `java.lang.String` | `getDocType()`             returns the current DOCTYPE, can be `null`. |
| `org.w3c.dom.Document` | `getDocument()`             returns the parsed DOM document. |
| `javax.xml.parsers.DocumentBuilderFactory` | `getFactory()`             returns the DocumentBuilderFactory. |
| `org.w3c.dom.Node` | `getNode(java.lang.String xpath)`             Returns the node represented by the XPath expression. |
| `java.lang.String` | `getRevision()`             Returns the revision string. |
| `java.lang.String` | `getRootNode()`             returns the current root node. |
| `boolean` | `getValidating()`             returns whether a validating parser is used. |
| `static void` | `main(java.lang.String[] args)`             for testing only. |
| `org.w3c.dom.Document` | `newDocument(java.lang.String docType, java.lang.String rootNode)`             creates a new Document with the given information. |
| `void` | `print()`             prints the current DOM document to standard out. |
| `org.w3c.dom.Document` | `read(java.io.File file)`             parses the given file and returns a DOM document. |
| `org.w3c.dom.Document` | `read(java.io.InputStream stream)`             parses the given stream and returns a DOM document. |
| `org.w3c.dom.Document` | `read(java.io.Reader reader)`             parses the given reader and returns a DOM document. |
| `org.w3c.dom.Document` | `read(java.lang.String xml)`             parses the given XML string (can be XML or a filename) and returns a DOM Document. |
| `void` | `setDocType(java.lang.String docType)`             sets the DOCTYPE-String to use in the XML output. |
| `void` | `setDocument(org.w3c.dom.Document newDocument)`             sets the DOM document to use. |
| `void` | `setRootNode(java.lang.String rootNode)`             sets the root node to use in the XML output. |
| `void` | `setValidating(boolean validating)`             sets whether to use a validating parser or not.  Note: this does clear the current DOM document! |
| `java.lang.String` | `toString()`             returns the current DOM document as XML-string. |
| `void` | `write(java.io.File file)`             writes the current DOM document into the given file. |
| `void` | `write(java.io.OutputStream stream)`             writes the current DOM document into the given stream. |
| `void` | `write(java.lang.String file)`             writes the current DOM document into the given file. |
| `void` | `write(java.io.Writer writer)`             writes the current DOM document into the given writer. |

**Methods inherited from class java.lang.Object**

`equals, getClass, hashCode, notify, notifyAll, wait, wait, wait`

**Field Detail**

### PI

```
public static final java.lang.String PI
```

:   the parsing instructions "<?xml version=\"1.0\" encoding=\"utf-8\"?>"
    (may not show up in Javadoc due to tags!).

    **See Also:**: Constant Field Values

---


### DTD\_DOCTYPE

```
public static final java.lang.String DTD_DOCTYPE
```

:   the DocType definition.

    **See Also:**: Constant Field Values

---


### DTD\_ELEMENT

```
public static final java.lang.String DTD_ELEMENT
```

:   the Element definition.

    **See Also:**: Constant Field Values

---


### DTD\_ATTLIST

```
public static final java.lang.String DTD_ATTLIST
```

:   the AttList definition.

    **See Also:**: Constant Field Values

---


### DTD\_OPTIONAL

```
public static final java.lang.String DTD_OPTIONAL
```

:   the optional marker.

    **See Also:**: Constant Field Values

---


### DTD\_AT\_LEAST\_ONE

```
public static final java.lang.String DTD_AT_LEAST_ONE
```

:   the at least one marker.

    **See Also:**: Constant Field Values

---


### DTD\_ZERO\_OR\_MORE

```
public static final java.lang.String DTD_ZERO_OR_MORE
```

:   the zero or more marker.

    **See Also:**: Constant Field Values

---


### DTD\_SEPARATOR

```
public static final java.lang.String DTD_SEPARATOR
```

:   the option separator.

    **See Also:**: Constant Field Values

---


### DTD\_CDATA

```
public static final java.lang.String DTD_CDATA
```

:   the CDATA placeholder.

    **See Also:**: Constant Field Values

---


### DTD\_ANY

```
public static final java.lang.String DTD_ANY
```

:   the ANY placeholder.

    **See Also:**: Constant Field Values

---


### DTD\_PCDATA

```
public static final java.lang.String DTD_PCDATA
```

:   the #PCDATA placeholder.

    **See Also:**: Constant Field Values

---


### DTD\_IMPLIED

```
public static final java.lang.String DTD_IMPLIED
```

:   the #IMPLIED placeholder.

    **See Also:**: Constant Field Values

---


### DTD\_REQUIRED

```
public static final java.lang.String DTD_REQUIRED
```

:   the #REQUIRED placeholder.

    **See Also:**: Constant Field Values

---


### ATT\_VERSION

```
public static final java.lang.String ATT_VERSION
```

:   the "version" attribute.

    **See Also:**: Constant Field Values

---


### ATT\_NAME

```
public static final java.lang.String ATT_NAME
```

:   the "name" attribute.

    **See Also:**: Constant Field Values

---


### VAL\_YES

```
public static final java.lang.String VAL_YES
```

:   the value "yes".

    **See Also:**: Constant Field Values

---


### VAL\_NO

```
public static final java.lang.String VAL_NO
```

:   the value "no".

    **See Also:**: Constant Field Values


**Constructor Detail**

### XMLDocument

```
public XMLDocument()
            throws java.lang.Exception
```

:   initializes the factory with non-validating parser.

    **Throws:**: `java.lang.Exception` - if the construction fails

---


### XMLDocument

```
public XMLDocument(java.lang.String xml)
            throws java.lang.Exception
```

:   Creates a new instance of XMLDocument.

    **Parameters:**: `xml` - the xml to parse (if "xml" is not found then it is considered a file) <DT**Throws:**: `java.lang.Exception` - if the construction of the DocumentBuilder fails **See Also:**: `setValidating(boolean)`

---


### XMLDocument

```
public XMLDocument(java.io.File file)
            throws java.lang.Exception
```

:   Creates a new instance of XMLDocument.

    **Parameters:**: `file` - the XML file to parse **Throws:**: `java.lang.Exception` - if the construction of the DocumentBuilder fails **See Also:**: `setValidating(boolean)`

---


### XMLDocument

```
public XMLDocument(java.io.InputStream stream)
            throws java.lang.Exception
```

:   Creates a new instance of XMLDocument.

    **Parameters:**: `stream` - the XML stream to parse **Throws:**: `java.lang.Exception` - if the construction of the DocumentBuilder fails **See Also:**: `setValidating(boolean)`

---


### XMLDocument

```
public XMLDocument(java.io.Reader reader)
            throws java.lang.Exception
```

:   Creates a new instance of XMLDocument.

    **Parameters:**: `reader` - the XML reader to parse **Throws:**: `java.lang.Exception` - if the construction of the DocumentBuilder fails **See Also:**: `setValidating(boolean)`


**Method Detail**

### getFactory

```
public javax.xml.parsers.DocumentBuilderFactory getFactory()
```

:   returns the DocumentBuilderFactory.

    :   **Returns:**: the DocumentBuilderFactory

---


### getBuilder

```
public javax.xml.parsers.DocumentBuilder getBuilder()
```

:   returns the DocumentBuilder.

    :   **Returns:**: the DocumentBuilder

---


### getValidating

```
public boolean getValidating()
```

:   returns whether a validating parser is used.

    :   **Returns:**: whether a validating parser is used

---


### setValidating

```
public void setValidating(boolean validating)
                   throws java.lang.Exception
```

:   sets whether to use a validating parser or not.  
    Note: this does clear the current DOM document!

    :   **Parameters:**: `validating` - whether to use a validating parser **Throws:**: `java.lang.Exception` - if the instantiating of the DocumentBuilder fails

---


### getDocument

```
public org.w3c.dom.Document getDocument()
```

:   returns the parsed DOM document.

    :   **Returns:**: the parsed DOM document

---


### setDocument

```
public void setDocument(org.w3c.dom.Document newDocument)
```

:   sets the DOM document to use.

    :   **Parameters:**: `newDocument` - the DOM document to use

---


### setDocType

```
public void setDocType(java.lang.String docType)
```

:   sets the DOCTYPE-String to use in the XML output. Performs NO checking!
    if it is `null` the DOCTYPE is omitted.

    :   **Parameters:**: `docType` - the DOCTYPE definition to use in XML output

---


### getDocType

```
public java.lang.String getDocType()
```

:   returns the current DOCTYPE, can be `null`.

    :   **Returns:**: the current DOCTYPE definition, can be `null`

---


### setRootNode

```
public void setRootNode(java.lang.String rootNode)
```

:   sets the root node to use in the XML output. Performs NO checking with
    DOCTYPE!

    :   **Parameters:**: `rootNode` - the root node to use in the XML output

---


### getRootNode

```
public java.lang.String getRootNode()
```

:   returns the current root node.

    :   **Returns:**: the current root node

---


### clear

```
public void clear()
```

:   sets up an empty DOM document, with the current DOCTYPE and root node.

    :   **See Also:**: `setRootNode(String)`, `setDocType(String)`

---


### newDocument

```
public org.w3c.dom.Document newDocument(java.lang.String docType,
                                        java.lang.String rootNode)
```

:   creates a new Document with the given information.

    :   **Parameters:**: `docType` - the DOCTYPE definition (no checking happens!), can be null: `rootNode` - the name of the root node (must correspond to the one given in `docType`) **Returns:**: returns the just created DOM document for convenience

---


### read

```
public org.w3c.dom.Document read(java.lang.String xml)
                          throws java.lang.Exception
```

:   parses the given XML string (can be XML or a filename) and returns a
    DOM Document.

    :   **Parameters:**: `xml` - the xml to parse (if "xml" is not found then it is considered a file) <DT**Returns:**: the parsed DOM document **Throws:**: `java.lang.Exception` - if something goes wrong with the parsing

---


### read

```
public org.w3c.dom.Document read(java.io.File file)
                          throws java.lang.Exception
```

:   parses the given file and returns a DOM document.

    :   **Parameters:**: `file` - the XML file to parse **Returns:**: the parsed DOM document **Throws:**: `java.lang.Exception` - if something goes wrong with the parsing

---


### read

```
public org.w3c.dom.Document read(java.io.InputStream stream)
                          throws java.lang.Exception
```

:   parses the given stream and returns a DOM document.

    :   **Parameters:**: `stream` - the XML stream to parse **Returns:**: the parsed DOM document **Throws:**: `java.lang.Exception` - if something goes wrong with the parsing

---


### read

```
public org.w3c.dom.Document read(java.io.Reader reader)
                          throws java.lang.Exception
```

:   parses the given reader and returns a DOM document.

    :   **Parameters:**: `reader` - the XML reader to parse **Returns:**: the parsed DOM document **Throws:**: `java.lang.Exception` - if something goes wrong with the parsing

---


### write

```
public void write(java.lang.String file)
           throws java.lang.Exception
```

:   writes the current DOM document into the given file.

    :   **Parameters:**: `file` - the filename to write to **Throws:**: `java.lang.Exception` - if something goes wrong with the parsing

---


### write

```
public void write(java.io.File file)
           throws java.lang.Exception
```

:   writes the current DOM document into the given file.

    :   **Parameters:**: `file` - the filename to write to **Throws:**: `java.lang.Exception` - if something goes wrong with the parsing

---


### write

```
public void write(java.io.OutputStream stream)
           throws java.lang.Exception
```

:   writes the current DOM document into the given stream.

    :   **Parameters:**: `stream` - the filename to write to **Throws:**: `java.lang.Exception` - if something goes wrong with the parsing

---


### write

```
public void write(java.io.Writer writer)
           throws java.lang.Exception
```

:   writes the current DOM document into the given writer.

    :   **Parameters:**: `writer` - the filename to write to **Throws:**: `java.lang.Exception` - if something goes wrong with the parsing

---


### getChildTags

```
public static java.util.Vector getChildTags(org.w3c.dom.Node parent)
```

:   returns all non tag-children from the given node.

    :   **Parameters:**: `parent` - the node to get the children from **Returns:**: a vector containing all the non-text children

---


### getChildTags

```
public static java.util.Vector getChildTags(org.w3c.dom.Node parent,
                                            java.lang.String name)
```

:   returns all non tag-children from the given node.

    :   **Parameters:**: `parent` - the node to get the children from: `name` - the name of the tags to return, "" for all **Returns:**: a vector containing all the non-text children

---


### findNodes

```
public org.w3c.dom.NodeList findNodes(java.lang.String xpath)
```

:   Returns the nodes that the given xpath expression will find in the
    document. Can return null if an error occurred.

    :   **Parameters:**: `xpath` - the XPath expression to run on the document **Returns:**: the nodelist

---


### getNode

```
public org.w3c.dom.Node getNode(java.lang.String xpath)
```

:   Returns the node represented by the XPath expression.
    Can return null if an error occurred.

    :   **Parameters:**: `xpath` - the XPath expression to run on the document **Returns:**: the node

---


### evalBoolean

```
public java.lang.Boolean evalBoolean(java.lang.String xpath)
```

:   Evaluates and returns the boolean result of the XPath expression.

    :   **Parameters:**: `xpath` - the expression to evaluate **Returns:**: the result of the evaluation, null in case of an error

---


### evalDouble

```
public java.lang.Double evalDouble(java.lang.String xpath)
```

:   Evaluates and returns the double result of the XPath expression.

    :   **Parameters:**: `xpath` - the expression to evaluate **Returns:**: the result of the evaluation, null in case of an error

---


### evalString

```
public java.lang.String evalString(java.lang.String xpath)
```

:   Evaluates and returns the boolean result of the XPath expression.

    :   **Parameters:**: `xpath` - the expression to evaluate **Returns:**: the result of the evaluation

---


### getContent

```
public static java.lang.String getContent(org.w3c.dom.Element node)
```

:   returns the text between the opening and closing tag of a node
    (performs a `trim()` on the result).

    :   **Parameters:**: `node` - the node to get the text from **Returns:**: the content of the given node

---


### print

```
public void print()
```

:   prints the current DOM document to standard out.

---


### toString

```
public java.lang.String toString()
```

:   returns the current DOM document as XML-string.

    :   **Overrides:**: `toString` in class `java.lang.Object`
    :   **Returns:**: the document as XML-string representation

---


### getRevision

```
public java.lang.String getRevision()
```

:   Returns the revision string.

    :   **Specified by:**: `getRevision` in interface `RevisionHandler`
    :   **Returns:**: the revision

---


### main

```
public static void main(java.lang.String[] args)
                 throws java.lang.Exception
```

:   for testing only. takes the name of an XML file as first arg, reads that
    file, prints it to stdout and if a second filename is given, writes the
    parsed document to that again.

    :   **Parameters:**: `args` - the commandline arguments **Throws:**: `java.lang.Exception` - if something goes wrong


---


|  |  |  |
| --- | --- | --- |
| **Overview** \| **Package** \| **Class** \| **Tree** \| **Deprecated** \| **Index** \| **Help** \| **Weka's home** | |  |
| **PREV CLASS**   **NEXT CLASS** | **FRAMES**    **NO FRAMES**     **All Classes** |  |
| SUMMARY: NESTED \| FIELD \| CONSTR \| METHOD | DETAIL: FIELD \| CONSTR \| METHOD |  |


---
